# Ultra Wide-Band Localization and SLAM: A Comparative Study for Mobile Robot Navigation

**DOI:** 10.3390/s110202035

**Published:** 2011-02-10

**Authors:** Marcelo J. Segura, Fernando A. Auat Cheein, Juan M. Toibero, Vicente Mut, Ricardo Carelli

**Affiliations:** Instituto de Automatica, National University of San Juan, Av. Libertador Gral. San Martin 1109 Oeste, J5400ARL San Juan, Argentina

**Keywords:** ultra wide-band, SLAM, mobile robots

## Abstract

In this work, a comparative study between an Ultra Wide-Band (UWB) localization system and a Simultaneous Localization and Mapping (SLAM) algorithm is presented. Due to its high bandwidth and short pulses length, UWB potentially allows great accuracy in range measurements based on Time of Arrival (TOA) estimation. SLAM algorithms recursively estimates the map of an environment and the pose (position and orientation) of a mobile robot within that environment. The comparative study presented here involves the performance analysis of implementing in parallel an UWB localization based system and a SLAM algorithm on a mobile robot navigating within an environment. Real time results as well as error analysis are also shown in this work.

## 1. Introduction

This article addresses the experimental comparison analysis between an ultra wide-band localization system (UWB) and a SLAM (Simultaneous Localization and Mapping) algorithm.

Indoor location systems have some problems such as the ability to locate objects exactly. This can be caused by a number of factors depending on the system being used. Each system has its advantages and its drawbacks. Some can provide a high degree of accuracy but are not suitable for manufacturing businesses, as they do not perform well in these conditions partly due to interference caused by other machinery. Cost is another factor as some localization systems can be very expensive to implement. Scalability is also another issue that requires investigation. However in order to evaluate these systems we must look at how the different systems operate, as well as their advantages and disadvantages. Ultra Wide Band technologies are often described as the next generation of real time location positioning systems. In the world today industry is becoming more competitive and any technology that can provide a competitive edge is welcome.

Industrial mobile robots, stock control and logistics in warehouses, mobility assistance for handicapped people or patient monitoring in hospitals are some scenarios that require accurate position estimation in indoor environments. Sensors based on ultrasound, lasers, cameras and Radio Frequency signals (RF) are often used for these applications. Radio Frequency sensors have a wide range of usage as the electromagnetic waves propagate through most typical environments. Impulse-based ultra wideband transceivers offer accurate ranging with low cost. However, one of the most significant obstacles in accurate ranging and positioning is the non-line of sight (NLOS) problem, which occurs in shadowed environments where a signal that propagates with a clear line of sight (LOS) may not be available. There are different wireless technologies, like WiFi [1], ZigBee [2], ultrasound [3], and others narrow band RF systems that have been proposed for indoor localization. Generally, these localization systems are based on low cost and low power sensors that measure received power or time of arrival. In the comparative study of Clarke *et al.* [4], it is shown that UWB is one of the best candidates for low cost accurate indoor localization. Commercial systems that use UWB sensors for indoor asset tracking are already available. For example, Zebra Enterprise Solutions [5] utilize time-difference-of-arrival (TDOA), and Ubisense [6] use a combination of TDOA and angle-of-arrival (AOA). The specified real-time accuracy of these systems is sub-15 cm with indoor operating ranges of over 50 m to 100 m.

In a previous work, a Mobile Robot Self-Localization systems using UWB technology was introduced [7]. The typical approach of commercial indoor localization systems consists of multiple base stations (BS) that receive an UWB signal from the tag to be localized. The received signals are processed by a central control unit or by the BS to finally estimate the tag or mobile robot position. In the mentioned work, a new approach for mobile robot localization was developed and it is implemented in this comparative study.

The SLAM algorithm applied on a mobile robot recursively estimates the pose—localization and orientation—of the vehicle and the elements of the environment—called map—while reducing errors associated with the estimation process [8,9]. Several algorithms have been proposed as solutions to the SLAM problem. The most widely used by the scientific community is the Extended Kalman filter (EKF) [8,10–12] solution and its derived filters, such as the Unscented Kalman filter (UKF) [12] and the Extended Information filter (EIF) [13,14]. In these filters, the SLAM system state, composed by the robot’s pose and the map of the environment, is modeled as a Gaussian random variable. Others solutions has also been implemented to solve the SLAM problem with high success, such as the case of the Particle filter (PF) [15], the *Graph-SLAM* [16] and the *FastSLAM* presented in [12].

Different SLAM algorithms solutions are presented to solve one or several issues associated with the SLAM process, such as the time consuming processing, the accuracy of the map, the successful closure of the loop, the integration of the SLAM algorithm with control laws to drive the vehicle motion and the modeling of different environments (dynamic, highly dynamic, static, structured, unstructured, *etc*.) [9,12]. Thus, for example, the EKF-SLAM presented in [10] extracted maps lines from structured environments, whereas [17] works on environments with point-based features (parameterized as range and bearing). The EKF has also been used in vision-based SLAM. Despite the easy implementation of the EKF-SLAM, its correction part demands high computation resources. To solve this, the EIF is used instead of the EKF [12].

In this article, an experimental comparison between the UWB localization method and the SLAM algorithm is performed. The UWB localization system and the SLAM algorithm are implemented in parallel on a mobile robot. The SLAM algorithm is implemented on an Extended Kalman filter (EKF) and extracts corners and lines—associated with walls—from the environment.

The comparison analysis involves the pros and cons of both methods when implemented on the mobile robot platform for navigation purposes. The experimental analysis includes covariances estimation evolution, accuracy of the localization methods, portability and feasibility of the SLAM and the UWB system, and error analysis. Finally, a comparative table is presented showing the advantages and disadvantages of both techniques presented in this work.

The article is organized as follows: Section 2 shows the general system implemented in this work; Section 3 introduces the UWB localization system; Section 4 presents the SLAM algorithm, the mobile robot and the map’s features model used in this work; Section 5 shows the experimental results of carrying out parallel experimentations of the UWB localization system and the SLAM algorithm. Section 6 concludes.

## 2. General System Architecture

**Figure 1.**
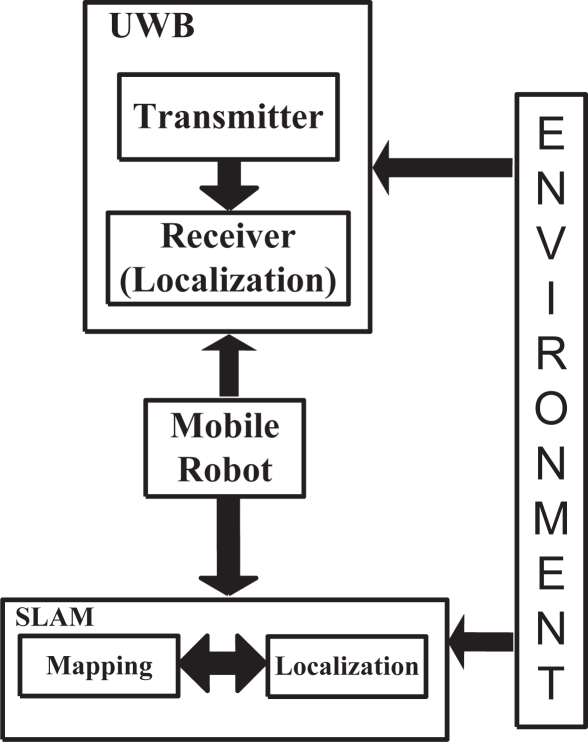
General System Architecture. The UWB localization system and the SLAM algorithm estimations are independent between them and both related to the same environment.

The system architecture of the comparative study between the UWB localization system and the SLAM algorithm is shown in Figure 1. Both localization systems (UWB and SLAM) are implemented in parallel and their localization estimations are independent from each other.

Given that the mobile robot has two independent localization systems implemented on it, the navigation of the vehicle should not rely on any of them [18] in order to avoid contradictory driving commands. Thus, the mobile robot motion is controlled by hand-joystick. This way, the mobile robot navigates within the same environment, following the same path for both localization methods.

The UWB localization system transmitters are located within the environments whereas the UWB localization system receiver is located on the mobile robot (see Figure 1). This scenario will be explained in detail in Section 3. On the other hand, the SLAM algorithm depends only on the exteroceptive sensors [18] of the mobile robot. UWB localization system estimates the position of the robot whereas the SLAM algorithm estimates both the position and the orientation of the mobile robot while it is navigating within the environment.

The following sections will show in detail each block of Figure 1.

## 3. Ultra Wide-Band Localization System

Generally, typical approaches of indoor localization systems consists of multiple base stations (BS) that receive an UWB signal from the robot to be localized. The received signals are processed by a central control unit or by the BS and finally estimate Mobile Robot (MR) position [19]. In mobile robot control application the position information must be sent back to the mobile vehicle, sometimes using a different communication system [20].

In this work, four synchronized anchor nodes are located in fixed known positions in the indoor environment. These anchor nodes transmit synchronized UWB pulses modulated as DBPSK (Differential Binary Phase Shift Keying), using a carrier of 3.5 GHz. In the current implementation, 2 ns pulses having a −10 dB bandwidth of approximately 1 GHz have been used. The mobile robot is equipped with an UWB receiver that estimates the robot localization measuring the Time Difference of Arrival (TDOA) between pairs of synchronized base stations. The UWB system used on this comparative study is defined as self-localization algorithm because the estimation algorithms run locally on the MR.

### 3.1. UWB Communication System and General Architecture

There are different UWB approaches that have been proposed for communication and localization systems like orthogonal frequency division multiplex [7], frequency hopping [19], chirp or direct-sequence spread spectrum modulation [20]. Nowadays, there is a general agreement that optimum receiver structures from conventional narrow-band communications systems are not feasible for low-power UWB communications. Currently there are different approaches to design sub-optimum, non-coherent schemes, based on energy detectors or differential detection. In our case, an UWB non-coherent system based on differential detection was implemented due to its simplicity and robustness, also because it allows to develop implementations with small power consumption.

The modulated Differential Binary Phase Shift Keying (DBPSK) signal in a complex form can be written as:

(1) $$s(t)=\sqrt{E_{b}}\sum_{k=-\infty }^{+\infty }(2\tilde{b}(k)-1)p(t-{\mathrm{kT}}_{f})$$

In (1), *b̃*(*k*) = *b*(*k*) ⊕ *b̃*(*k* − 1), *b̃*(*k*) ∈ {0, 1}. ⊕ is the logical *or* operator, *b̃*(*k*) are the differential encoded pulses, and *p*(*t*) is the selected pulse shape (*E_b_* is the bit energy). An IR-UWB system transmits each information symbol over a time interval of *T_s_* seconds, which consists of *N_f_* frames of length *T_f_* and the resulted symbol length is *T_s_* = *N_f_* × T*_f_*. In each frame, a short pulse *p*(*t*) of *T_p_* = 2ns is transmitted with the selected shape.

The mobile robot must identify multiple signals that come from different base stations. In order to differentiate each BS, Direct Sequence (DS) Gold spreading codes are differentially encoded prior to the modulation. The code length 7, which is the smallest Gold code, was implemented for reducing acquisition and processing time. It is worth noting that better accuracy could be achieved if longer codes were implemented. If the base stations transmit at the same time, inter-pulse interference takes place, hence a simple time division multiple access (TDMA) was implemented. The final DS-UWB DBPSK transmitted signal can be written as:

(2) $$x_{u}(t)=\sqrt{E_{b}}\sum_{k=-\infty }^{+\infty }(2\tilde{b}(k)-1)p(t-{\mathrm{kT}}_{f}-{\mathrm{uT}}_{s})$$

In (2), *x_u_*(*t*) is the transmitted signal by the base station *BS_u_*, *u* = 0, 1, ...,*N_BS_* − 1 and *N_BS_* is the number of base stations, in our case *N_BS_* = 4. The UWB transmitter use an RF switch to distribute the corresponding signal to each antenna.

Regarding the receiver, differential demodulation is necessary to compare the phase of the previous pulse with the phase of the current pulse. To do this, the delay needs to be as large as the frame time *T_f_*. The receiver accuracy is related with the capacity to make a precise delay. Since it is difficult to make an accurate analog delay, the authors used the concept of Software Defined Radio (SDR), in which the analog to digital converter (ADC) is placed as close as possible to the antenna.

### 3.2. UWB Localization Algorithm

**Figure 2.**
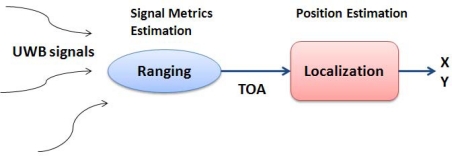
Two step localization scheme.

The localization algorithm is based on *two step positioning* approach. In the first step of a two-step localization system, signal parameters are estimated for ranging purpose, in our case the time-of-arrival (TOA). Then, in the second step, the target node position is estimated based on the signal parameters obtained from the first step using adequate positioning algorithms. A block diagram of two-step localization system is illustrated in Figure 2.

**Figure 3.**
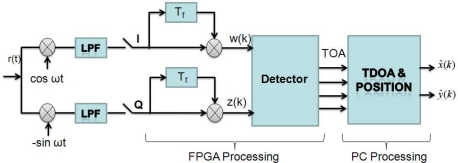
Block diagram of the implemented receiver structure, Time of Arrival, and positions estimation algorithms.

**Figure 4.**
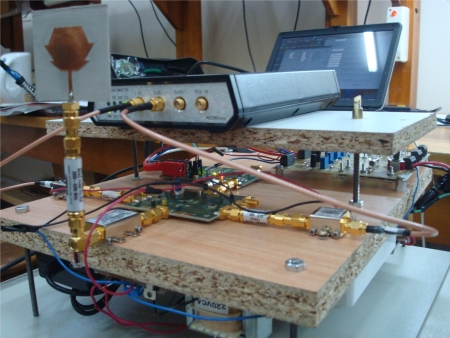
Receiver implemented with off-the-shelf components.

For ranging purpose the received signal *r*(*t*) is correlated with a delayed signal coming from the same BS. This correlation improves the signal to noise ratio (SNR) because the signals are scattered by the same objects or building furniture and modified by the same communication channel, so they are highly correlated. In the implemented receiver, a high sampling frequency ADC is placed after the low pass filters and the signal detection is made on digital domain using a Field Programmable Gate Array (FPGA) (Figure 3). The real receiver that was mounted over the MR is shown in Figure 4.

**Figure 5.**
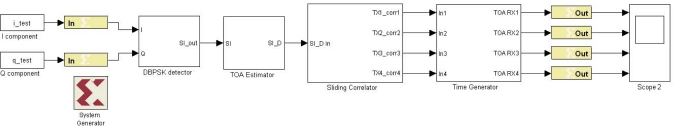
Detection and TOA estimation on FPGA.

The TOA estimation is implemented on the FPGA due to its parallel processing capabilities, which permits to reduce the detection time. The implementation was done using the System Generator tool from **MatLab**, as Figure 5 shows.

The DBPSK detector block in Figure 5 computes the correlation with a delayed version of the incoming in-phase (*I*) and quadrature (*Q*) signal components. The recovered signal *SI_out_* is the input to the following block (TOA Estimator) which estimates the TOA using an adaptive threshold concept [20]. A threshold-based TOA algorithm selects the time at which the signal “SI” crosses an established threshold. The estimation accuracy depends on the threshold selection. If the threshold is low, the probability of detecting a peak due to noise increase, and it is defined as false alarm or early detection. In contrast, if the threshold is high, probability of detecting a signal that arrives later than the direct path increases, and this is called miss detection. An important issue is how to select a dynamic threshold that works well under different SNR and LOS conditions. This problem was solved by the dynamic threshold algorithm proposed in [20]. At the output of TOA estimator block, the signal is regenerated using the times estimated by the threshold algorithm.

The Sliding correlator block receives a digital recovered signal that will be cross-correlated against each Gold code templates. The output of this block is compound by a set of signals whose peaks take place when the code template matches the received signal. Next, the Time Generator block estimates the peaks time and generates the TOA estimations corresponding to each transmitter. The detailed ranging algorithm runs on real time over the FPGA. The required processing time depends on the code length and the number of BS. In our case the ADC capture 8192 samples @ 1.5 Gsps, which means 5.4 *μ*s for acquisition and detection since that the developed algorithm can run on line in the FPGA.

At the mobile robot, the estimation of the ranges between base stations and robot’s current position (see Equation 3) is performed by multiplying the estimated time difference with the radio speed (4). These ranges produce a set of hyperbolas and their intersections determine the MR position. In a 2-D position localization system, the base station position is expressed as (*X_bs_*, *Y_bs_*) and the mobile robot location is (*x_r_*, *y_r_*).

(3) $$R_{i}=\sqrt{{\left(X_{\mathrm{bs}}-x_{r}\right)}^{2}+{\left(Y_{\mathrm{bs}}-y_{r}\right)}^{2}}$$

The range difference between the base stations is:

(4) $$\begin{array}{c} R_{i,1}=c\tau_{i,1}=R_{i}-R_{1}\\ R_{i,1}=\sqrt{{\left(X_{i}-x_{r}\right)}^{2}+{\left(Y_{i}-y_{r}\right)}^{2}}-\sqrt{{\left(X_{1}-x_{r}\right)}^{2}+{\left(Y_{1}-y_{r}\right)}^{2}} \end{array}$$

In order to solve the set of non-linear equations, a linear constrained least square algorithm that applies the technique of Lagrange multipliers to solve the minimization problem proposed by [21] is implemented. The localization algorithm runs on a PC located over the mobile robot. The processing time takes 300 ms on a Core2Duo @ 1.66 GHz. Majority of this time is due to USB handshake between PC and ADC-FPGA board.

## 4. SLAM Algorithm

The SLAM (Simultaneous Localization and Mapping) algorithm recursively estimates the pose—position and orientation—of the mobile robot within the environment while mapping the same environment [8,9].

The SLAM algorithm implemented in this work is solved by an Extended Kalman filter (EKF). The SLAM system state is composed by the vehicle estimated pose—position and orientation—and the features extracted from the environment, which are known as the *map of the environment*. The features extracted from the environment correspond to corners (concave and convex) and lines (associated with walls). For visualization and map reconstruction purposes, a secondary map is maintained. This secondary map stores the beginning and ending points of the segments associated with the lines of the environment. Thus, the secondary map allows finite walls representation. The secondary map is updated and corrected according to the feature correction in the EKF-SLAM system state, and if a new feature is added to that system state, it is also added in the secondary map [22]. Equations (5) and (6) show the system state structure and its covariance matrix. All elements of the SLAM system state are referenced to a global coordinate system.

(5) $${\hat{\xi }}_{t}=\left[\begin{array}{c} {\hat{\xi }}_{v,t}\\ {\hat{\xi }}_{m,t} \end{array}\right]$$

(6) $$P_{t}=\left[\begin{array}{cc} P_{\mathrm{vv},t} & P_{\mathrm{vm},t}\\ P_{\mathrm{vm},t}^{T} & P_{\mathrm{mm},t} \end{array}\right]$$

In Equation (5), *ξ̂_t_* is the SLAM system state; *ξ̂_v,t_* = [*x̂_t_* *ŷ_t_* *θ̂_t_*] is the estimated pose of the vehicle, where *x̂_t_* and *ŷ_t_* represent the global position of the agent within the environment and *θ̂_t_* its orientation; *ξ̂_m,t_* represents the map of the environment and it is composed by parameters that define both lines and corners (corners are defined in the Cartesian space and lines in the polar space, as will be shown in Section 4.2). The order in which lines and corners appear in *ξ̂_m,t_* depends on the moment they were detected. *P_t_* is the covariance matrix associated with the SLAM system state; *P_vv,t_* is the covariance of the vehicle’s pose and *P_mm,t_* is the covariance of the map. *P_vm,t_* and $P_{\mathrm{mv},t}^{T}$ are cross-correlation matrices (between the vehicle and the map).

The covariance matrix initialization techniques and the EKF definition can be found in [8,9]. The EKF is represented in Equation (7). All variables involved in the estimation process are considered as Gaussian random variables.

(7) $$\{\begin{array}{l} {\hat{\xi }}_{t}^{-}=f({\hat{\xi }}_{t},u_{t})\\ P_{t}^{-}=A_{t}P_{t-1}A_{t}^{T}+W_{t}Q_{t-1}W_{t}^{T}\\ K_{t}=P_{t}^{-}H_{t}^{T}{\left(H_{t}P_{t}^{-}H_{t}^{T}+R_{t}\right)}^{-1}\\ {\hat{\xi }}_{t}=\xi_{t}^{-}+K_{t}(z_{t}-h({\hat{\xi }}_{t}^{-}))\\ P_{t}=(I-K_{t}H_{t})P_{t}^{-}. \end{array}$$

In Equation (7), ${\hat{\xi }}_{t}^{-}$ is the predicted state of the system at time *t; u_t_* is the input control commands and *ξ̂_t_* is the corrected state at time *t; f* describes the motion of the elements of *ξ̂*. $P_{t}^{-}$ and *P_t_* are the predicted and corrected covariance matrices respectively at time *t; A_t_* is the Jacobian of *f* with respect to the SLAM system state and *Q_t_* is the covariance matrix of the noise associated to the process, whereas *W_t_* is its Jacobian matrix; *K_t_* is the Kalman gain at time *t; H_t_* is the Jacobian matrix of the measurement model (*h*) and *R_t_* is the covariance matrix of the actual measurement(*z_t_*). The term ( $z_{t}-h({\hat{\xi }}_{t}^{-})$) is called the innovation vector [12] and takes place when the data association procedure has reached an appropriate matching between the observed feature and the predicted one ( $h({\hat{\xi }}_{t}^{-})$). Both the process model (*f*) and the observation model are non-linear expressions. Further information concerning the EKF-SLAM can be found in [22].

In this work, the sequential EKF was implemented in order to reduce computational costs. The sequential EKF-SLAM is based on the iterative calculation of the correction stage (SLAM system state and covariance matrix) for each feature with correct association, see [12]. The prediction stage remains as stated in Equation (7).

The general form of the correction stage of the classical sequential EKF-SLAM algorithm [12] is summarized in the algorithm shown in Algorithm 1. Sentences (3) to (9) describe the *for* loop of the correction stage of the algorithm. For every feature with correct association (sentence (2)), the *for* loop is executed. Sentence (4) shows the Kalman gain calculation; sentence (5) is the correction of the SLAM system state, whereas sentence (6) is the correction of the covariance matrix of the SLAM algorithm. In sentence (7), the current feature is deleted from the set of features with correct association (*M_t_*). In the next iteration, the next predicted SLAM system state and covariance matrix are the last corrected SLAM system state and covariance matrix respectively, as noted in sentence (8).

Further information concerning the EKF-SLAM implemented in this work can be found in [23].

### 4.1. Mobile Robot

**Figure 6.**
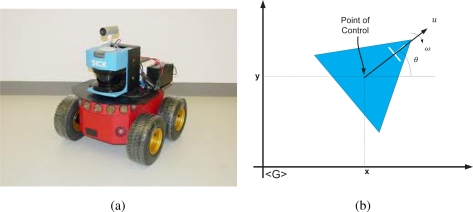
Mobile robot unicycle non-holonomic type, Pioneer 3AT used in this work. **(a)** shows a picture of the vehicle. **(b)** shows a graphic representation of the kinematic model of the vehicle.

The mobile robot used during the experimentation is a Pioneer 3AT built by ActivMedia. The Pioneer 3AT is an unicycle like non-holonomic mobile robot. The vehicle has a range sensor laser, built by SICK, incorporated on it that acquires 181 measurements between 0 and 180 degrees in a range of 32 m. Figure 6(a) shows a picture of the mobile robot used. The mobile robot kinematics equation are shown in Equation (8), whereas Figure 6(b) shows a graphic representation of the kinematic model of the robot.

**Algorithm 1:** Algorithm of the correction stage of the Sequential EKF-SLAM.

1: Let *N_t_* be set of the observed features 2: Let *M_t_* ⊆ *N_t_* be the set of features with correct association 3: **for** *j* = 1 to ⧣*M_t_* **do** 4: $K_{t,j}=P_{t,j}^{-}H_{t,j}^{T}{\left(H_{t,j}P_{t,j}^{-}H_{t,j}^{T}+R_{t,j}\right)}^{-1}$ 5: $\xi_{t,j}=\xi_{t,j}^{-}+K_{t,j}(z_{j}-h({\hat{\xi }}_{t,j}^{-}))$ 6: $P_{t,j}=(I-K_{t,j}H_{t,j}))P_{t,j}^{-}$ 7: *M_t,j_* = *M_t,j_* − {*z_j_*} 8: $P_{t,j}^{-}:=P_{t,j};{\hat{\xi }}_{t,j}^{-}={\hat{\xi }}_{t,j}$ 9: **end for**

(8) $${\left[\begin{array}{c} x_{t}\\ y_{t}\\ \theta_{t} \end{array}\right]}_{G}=\left[\begin{array}{c} x_{t-1}\\ y_{t-1}\\ \theta_{t-1} \end{array}\right]+\Delta t\;\left[\begin{array}{cc} \text{cos}(\theta_{t-1}) & 0\\ \text{sin}(\theta_{t-1}) & 0\\ 0 & 1 \end{array}\right]\;\left[\begin{array}{c} u_{t}\\ \omega_{t} \end{array}\right]+\Phi_{t}$$

In Equation (8), *x_t_*, *y_t_* and *θ_t_* are the coordinates of the point of control of the vehicle in Figure 6(b); Φ*_t_* is the Gaussian noise associated with the vehicle’s model; *u_t_* and *ω_t_* are the linear and the angular velocities respectively, generated by the control strategy. In this work, the mobile robot control commands were generated by means of a hand-joystick; Δ*t* is the sampling time and the suffix *G* implies that *x_t_*, *y_t_* and *θ_t_* (the pose of the vehicle) are expressed in a global reference frame of the environment [23].

### 4.2. Features of the Environment

**Figure 7.**
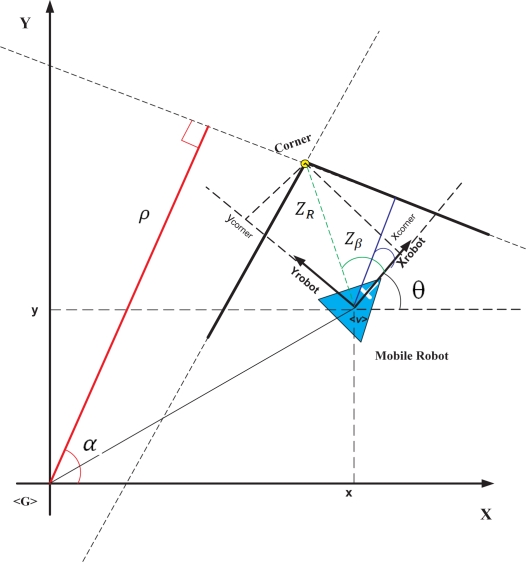
Graphic representation of line and corners extracted from the environment.

The models of the features of the environment (corners and lines) are shown in Equations (9) and (10). Figure 7 shows the graphical interpretation of the variables in Equations (9) and (10).

(9) $$\begin{array}{c} z_{\mathrm{corner}}(k)=h_{i}[{\hat{\xi }}_{v,t},w(k)]=\left[\begin{array}{c} z_{R}\\ z_{\beta } \end{array}\right]=\\ \left[\begin{array}{c} \sqrt{{\left({\hat{x}}_{t}(k)-x_{\mathrm{corner}}\right)}^{2}+{\left({\hat{y}}_{t}(k)-y_{\mathrm{corner}}\right)}^{2}}\\ \text{arctan}\;\frac{{\hat{y}}_{t}(k)-y_{\mathrm{corner}}}{{\hat{x}}_{t}-x_{\mathrm{corner}}}-{\hat{\theta }}_{t}(k) \end{array}\right]+\\ +\;\left[\begin{array}{c} w_{R}\\ w_{\beta } \end{array}\right] \end{array}$$

(10) $$\begin{array}{c} z_{\mathrm{line}}(k)=h_{i}[{\hat{\xi }}_{v,t},w(k)]=\\ \left[\begin{array}{c} Z_{\rho }\\ Z_{\alpha } \end{array}\right]=\left[\begin{array}{c} r-{\hat{x}}_{t}(k)\;\text{cos}(\alpha )-{\hat{y}}_{t}(k)\;\text{sin}(\alpha )\\ \alpha -{\hat{\theta }}_{t}(k) \end{array}\right]\\ +\;\left[\begin{array}{c} w_{\rho }\\ w_{\alpha } \end{array}\right] \end{array}$$

In Equations (9) and (10), *w_ρ_*, *w_α_*, *w_R_*, *w_β_* are additive Gaussian noise associated with the measurement. Further information concerning the line’s modeling can be found in [10].

## 5. Experimental Results

**Figure 8.**
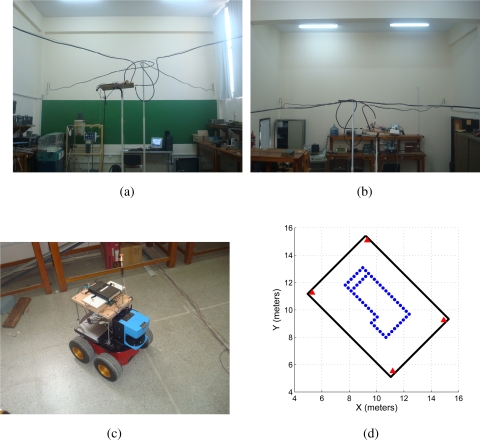
Environment disposition. **(a,b)** shows the four UWB transmitter antennas located on each wall of the environment, near their respective corner. **(c)** shows the Pioneer 3AT with both the UWB receiver antenna and the laser SICK, used by the SLAM algorithm. **(d)** shows the environment with the landmarks referenced to an arbitrary reference frame. The solid black lines represent walls of the environment, the solid red triangles are the UWB transmitter antennas and the solid blue dots are the landmarks on the floor of the environment.

The comparison experiments were carried out at the facilities of the Engineering Department of the National University of San Juan, Argentina. The environment was a static non-structured environment. Figure 8(a,b) shows two pictures of the four antennas located near the four walls intersections of the environment. They were located at a high of 2.45 m from the floor. The UWB receiver antenna was located at the mobile robot, at a high of 0.835 m from the floor. Figure 8(c) shows the mobile robot used (the Pioneer 3AT) with the laser SICK and the UWB receiver antenna on it. On the other hand, Figure 8(d) shows several landmarks intentionally located over the environment’s floor. The relative position of each landmark with respect to the transmitter antennas (see Figure 8(a,b)) is known. Thus, it is possible to reference the environment to any coordinate system [24]. Figure 8(d) shows the walls of the environment (solid black segments), the disposition of the transmitter antennas within it (solid red triangles) and the landmarks on the floor of the environment (solid blue dots).

### 5.1. UWB Localization

In order to determine the performance of the localization systems shown in Figure 1, the mobile robot is positioned at [*x y*]*^T^* = [10.5 8.2]*^T^* meters within the environment shown in Figure 8(d).

**Figure 9.**
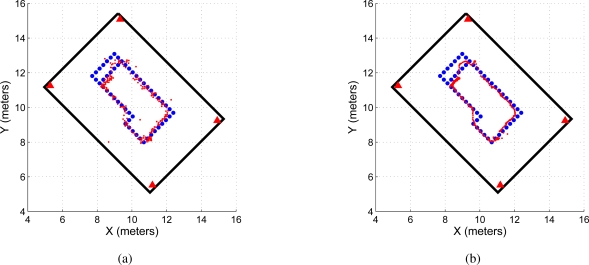
UWB localization measurements. **(a)** shows raw UWB localization data within the environment in which the mobile robot is navigating. **(b)** shows the UWB localization data after applying an averaging filter of five past samples. As it can be seen, data dispersion is reduced. Solid black segments are associated with walls of the environment; solid red triangles represent the UWB transmitter antennas’ locations; solid blue dots are intentionally located landmarks at the floor of the environment and solid red dots are the estimated localization measurements of the robot’s positions given by the UWB system.

With the purpose of avoiding disperse measurements –as the ones shown, e.g., at positions [*x y*]*^T^* = {[8.65 8]*^T^*, [8.44 12.8]*^T^*, [10.5 9.3]*^T^* } in Figure 9(a), a five samples average filtering is applied. This filter is an sliding window that averages five samples at a time, as shown in Equation (11).

(11) y[n]=(y[n]+y[n−1]+y[n−2]+y[n−3]+y[n−4])/5

In Equation (11), *y* is the localization measurement at instant *n*. Five past samples are needed in order to smooth the current measurement. Thus, only the first five localization measurement from the localization process are lost, which correspond to the robot at its initial position. Figure 9(b) shows the filtered measurements when reconstructing the traveled path of the mobile robot. As it can be seen, data dispersion is reduced.

### 5.2. EKF-SLAM: Mapping and Localization Results

**Figure 10.**
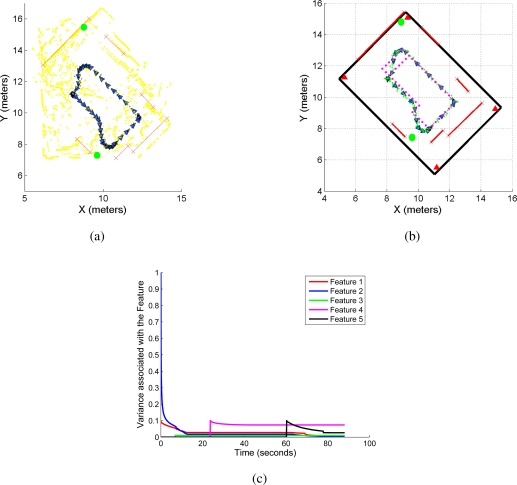
EKF-SLAM results. **(a)** shows the map reconstruction of the environment. The solid red segments are associated with line-features extracted by the SLAM algorithm; solid green circles are associated with corners; yellow points are raw laser data and solid green dots represent the path traveled by the mobile robot estimated by the SLAM algorithm. **(b)** shows the map obtained by the SLAM juxtaposed with the real map of the environment and the floor-landmarks. **(c)** shows the variance evolution of five features extracted from the SLAM system state, see Equation (5).

During the mobile robot navigation shown in Figure 9(a,b), the EKF-SLAM algorithm was running in parallel to the UWB localization system as stated in Section 2. Figure 10(a) shows the map reconstruction and the localization of the mobile robot within the navigated environment. The SLAM algorithm is performed on line based on the features extraction by the range sensor laser. In Figure 10(a), the yellow points are raw laser data, the solid red segments are associated with the line-features extracted from the environment and the solid green circles are associated with corners. The estimated path is represented by solid grey dots.

On the other hand, Figure 10(b) shows the mapped environment compared with its real geometric reconstruction. The solid black lines are the walls of the environment whereas the solid red triangles represent the UWB transmitter antennas; the solid magenta dots are the landmarks on the floor of the navigated environment whereas the solid green dots are the estimated path by the SLAM algorithm. The solid red segments and the solid green circles are the features extracted by the SLAM algorithm.

Figure 10(c) shows the variance evolution associated with five features extracted from the environment by the SLAM algorithm. As it can be seen, the variance of the features gradually decreases as established in [23,25], proving that the SLAM algorithm has consistently estimated the map of the environment after closing the loop (re-observation of the first extracted features [12]).

### 5.3. UWB vs. EKF-SLAM: Discussion

As stated in Section 2, the UWB localization system and the SLAM algorithm were implemented in parallel. Thus, the UWB localization and the SLAM estimation were performed during the same path traveled by the mobile robot. The SLAM maximum sampling time was 0.2 s whereas the UWB localization method sampling time was 0.3 s. In this section the advantages and disadvantages of both localization methods will be shown.

**Figure 11.**
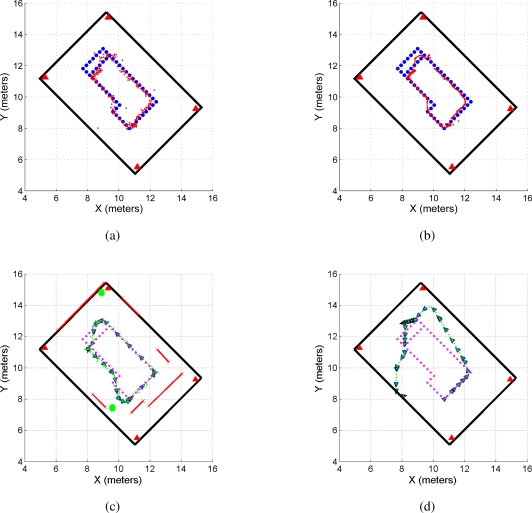
Path estimated by the localization system. **(a)** shows the path obtained by the UWB localization system without filtering data; **(b)** shows the path generated by the UWB localization system applying the filtering criterion shown in Equation (11). In both figures, the dotted red line is the path estimated by the UWB localization system. **(c)** shows the path estimated by the SLAM algorithm (dotted green line) and **(d)** shows the path estimated by the odometric data of the mobile robot. As it can be seen, the odometric data becomes inconsistent with the environment information.

Figure 11(a) shows the path obtained by the UWB localization method (dotted red line) also shown in Figure 9(a), whereas Figure 11(b) shows the UWB filtered path (in dotted red line) also shown in Figure 9(b). On the other hand, Figure 11(c) shows the path estimated by the SLAM algorithm (dotted green line) and Figure 11(d) shows the path estimated by the odometric data of the mobile robot [18] (in green dotted line). By inspection is possible to observe the following:

- The mobile robot path estimated by the odometric data (Figure 11(d)) is inconsistent with the environment. The path crosses through a wall of the environment. Odometric data is highly noisy [12] and cannot be used as a single localization measure.
- The UWB filtered path (Figure 11(b)) is smoother than the non-filtered UWB path (Figure 11(a)). Also, Figure 11(a) shows a more dispersed path when compared with Figure 11(b,c).
- The SLAM estimated path shows the smoothest path when compared with the UWB estimated paths.

**Figure 12.**
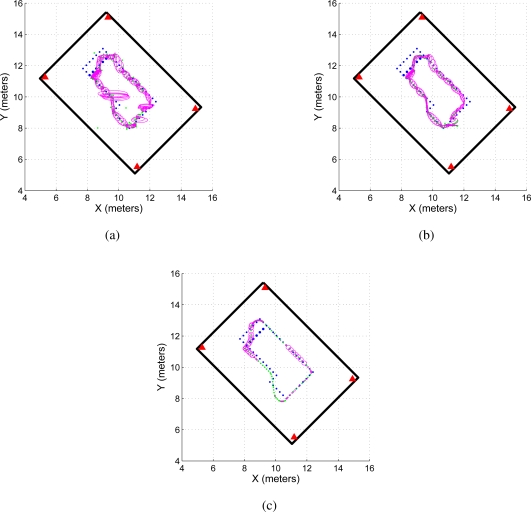
Covariance ellipses of the estimated paths. **(a)** shows the covariance ellipse of the UWB localization system without the filtering stage shown in Equation (11). Figure 12(b) shows the case for the filtered UWB data. As it can be seen, **(a)** is more disperse than **(b)**. **(c)** shows the covariance ellipses associated with the SLAM algorithm estimation.

In addition, Figure 12(a,b,c) shows the covariance ellipses associated with the UWB paths and the SLAM’s estimated path. For the path estimated by the SLAM algorithm, the covariance ellipses are obtained based on the covariance evolution of the SLAM system state along the experimentation [12]. For the UWB paths (Figure 12(a,b)), the covariance ellipses are generated based on successive measurements of the robot’s position before the execution of a control command (the robot remains still for several sampling times, acquiring UWB localization data). For example, if the robot is positioned at point *P*_1_ within the map, it acquires several UWB measurements at that point until it moves to point *P*_2_. In *P*_2_, the system acquires again several measurements. Those measurements associated with a same position are then used to build the uncertainty ellipse associated with a given position of the mobile robot.

As it can be seen, the path estimated by the SLAM algorithm (Figure 12(c)) shows a better path—from a covariance perspective—than the path obtained by the UWB localization system. Given the data dispersion associated with the UWB non-filtered localization method (Figure 12(a)), the covariance ellipses associated with it shows a bigger area than the ellipses of Figure 12(b) for the same robot’s position. Generally, the covariance increment happens due to non-line of sight conditions. As is possible to see in 12(a), big dispersion takes place at positions in which one of the antennas is totally blocked.

**Figure 13.**
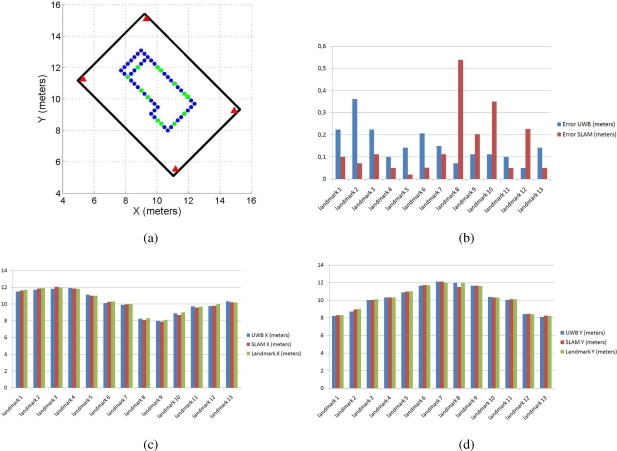
Error of the estimated paths. **(a)** shows the landmarks in green-dot points through where the mobile robot has passed during the experiment. **(b)** shows the norm of the error between the landmarks’ true position and the position of the mobile robot estimated by both the UWB localization method and the SLAM algorithm when the vehicle is positioned over such landmarks. **(c)** shows the errors in the *x* coordinate of the of the robot’s position estimation based on both the UWB localization method and the SLAM algorithm, when the vehicle is positioned on the landmarks shown in **(a)**. **(d)** shows the case for the *y*–coordinate.

Finally, the paths obtained by the UWB filtered localization system and the SLAM algorithm are compared with the landmarks located on the floor of the environment. As stated in Section 5, the position of the landmarks within the environment is previously known. The landmarks over which the mobile robot has passed are also known (they are shown in green-dot points in Figure 13(a)). Thus, Figure 13(b) shows the norm of the error between the landmarks’ true localization within the environment (shown in Figure 13(a)) and both the UWB localization estimation and the SLAM algorithm mobile robot’ pose estimation. Figure 13(c) and 13(d) show the error of the estimated position of the mobile robot based on both the UWB localization method and the SLAM algorithm for each coordinate. The estimation errors shown in Figure 13(c) and 13(d) are compared with respect to the true landmark position within the environment. Also, only the landmarks through which the mobile robot has passed (see Figure 13(a)) are considered for the calculation of the positioning errors. As it can be seen in Figure 13(c) and 13(d), both localization methods have shown similar errors during the experimentation.

Although both localization methods (UWB based and SLAM based) have shown similar results (errors and performance), there are important differences between them. For example, if an extra robot is added to the environment, the UWB localization based will require only a new UWB receiver, whereas the SLAM algorithm will require a new range sensor laser for this mobile robot and this SLAM algorithm. Also, the UWB localization system is restricted to the environment where the UWB transmitter antennas are located, whereas the SLAM algorithm is independent of the environment and human intervention. The SLAM algorithm is restricted by the nature of the features to be extracted from the environment (in this work, lines and corners), whereas the UWB localization system is independent of the elements of such environment (it could be either dynamic or static). Finally, as stated in [12], the SLAM algorithm needs to close the navigation loop in order to reduce the accumulative estimation errors whereas the UWB filtered localization method has a non-accumulative error. The advantages and disadvantages of both localization methods are summarized in Table 1.

## 6. Conclusions

The experimental results show that both localization systems can be used to localize mobile robots in indoor environments with high accuracy. The UWB localization system has greater variance compared with SLAM but the accuracy of both systems is similar. The main advantage of SLAM systems is that navigation is not restricted by the environment. In the UWB case, more transmitting antennas are needed in order to increase the coverage area. On the other hand, if the UWB receiver and transmitter will be integrated on the same chip, the mentioned problem could be solved. Furthermore, a low cost radar type sensor could locate the robot and map the environment as SLAM does. It is important to mention that the localization error is not cumulative on UWB localization system, because the proposed scheme gives an absolute result. However, the SLAM localization system must close the navigated loop in order to get the shown results. Finally, the accuracy could be improved and the variance could be bounded if more UWB pulses are acquired per position estimation. On the current UWB localization system, only one transmitted symbol is processed for position estimation, due to time constrains. If the TOA estimation and position algorithms were implemented on the Field Programmable Gate Array, better results could be reached.

## Figures and Tables

**Table 1.** Comparisons between the UWB localization system and SLAM algorithm.

UWB filtered localization system	SLAM algorithm

Depends on the UWB antennas disposition on the environment	Can map and localize in several maps of different sizes
An extra robot requires an extra UWB receiver	An extra robot requires a new range sensor appropriate for the SLAM algorithm
The localization error is not accumulative	The SLAM system state estimation error is accumulative until the SLAM closes the loop within the navigated environment (the mobile robot re-sees past features)
Concerns only localization problems	Acquires a map based on the features detection procedure incorporated on the SLAM algorithm
Features independent. The UWB localization system is not dependent on the nature of the features present within the environment	Depends on the detection and extraction of the determined features from the environment. If the SLAM algorithm does not extract any feature from the environment, then the localization errors are not minimized
Estimation does not turn into inconsistence	Due to bad features matching, the SLAM algorithm could turn into inconsistence
High dispersion of unfiltered data	Consistent estimation of the mobile robot navigated path
Covariance error remains bounded	Covariance error is minimized
Mobile robot autonomy is restricted to the sensed environment	Mobile robot autonomy is potentiated by SLAM [26]

## References

[1] Ladd A.M., Bekris E.K.E., Rudys A.P., Wallach D.S., Kavraki L.E. (2004). On the feasibility of using wireless Ethernet for indoor localization. IEEE T. Robotic Autom.

[2] Blumenthal J., Grossmann R., Golatowski F., Timmermann D. Weighted centroid localization in Zigbee-based sensor networks.

[3] Priyantha N., Chakraborty A., Balakrishnan H. The cricket location-support system.

[4] Clarke D., Park A. Active-RFID system accuracy and its implications for clinical applications.

[5] Zebra Enterprise Solutions http://zes.zebra.com/technologies/location/ultra-wideband.jsp/.

[6] Ubisense, Cambridge, CB4 1DL. UK. Available online: http://www.ubisense.net/en/products/precise-real-time-location.html (accessed on 29 January 2011).

[7] Segura M. J., Mut V., Patio H. Mobile robot self-localization system using IR-UWB sensor in indoor environments.

[8] Durrant-Whyte H., Bailey T. (2006). Simultaneous Localization and Mapping (SLAM): Part I Essential Algorithms. IEEE Robot. Autom. Mag.

[9] Durrant-Whyte H., Bailey T. (2006). Simultaneous Localization and Mapping (SLAM): Part II State of the Art. IEEE Robot. Autom. Mag.

[10] Garulli A., Giannitrapani A., Rossi A., Vicino A. Mobile robot SLAM for line-based environment representation.

[11] Tamjidi A., Taghirad H.D., Aghamohammadi A. On the consistency of EKF-SLAM: Focusing on the observation models.

[12] Thrun S, burgard W, Fox D (2005). Probabilistic Robotics.

[13] Cadena C., Neira J. SLAM in O(log n) with the Combined Kalman—Information filter.

[14] Yufeng L., Thrun S. Results for outdoor-SLAM using sparse extended information filters.

[15] Nosan K., Beom-Hee L., Yokoi K. Result representation of Rao-Blackwellized particle filtering for SLAM.

[16] Guo R., Sun F., Yua J. ICP based on Polar Point Matching with application to Graph-SLAM.

[17] Bailey T., Nieto J., Guivant J., Stevens M., Nebot E. Consistency of the EKF-SLAM Algorithm.

[18] Siegwart R., Nourbakhsh I. (2004). Autonomous Mobile Robots.

[19] Krishnan S., Sharma P., Guoping Z., Woon H. A UWB based localization system for indoor robot navigation ultra-wideband.

[20] Schroeder J., Galler S., Kyamakya K. A low-cost experimental ultra-wideband positioning system.

[21] Public Safety Communication Europe, D.4.1.1. (2006). Public Report EUROPCOM (Emergency Ultrawideband Radio for Positioning and Communications).

[22] Auat Cheein F., De la Cruz C., Carelli R., Bastos Filho T. F. Solution to a door crossing problem for an autonomous wheelchair.

[23] Auat Cheein F., Scaglia G., di Sciascio F., Carelli R. (2009). Feature selection criteria for real time EKF-SLAM algorithm. Int. J. Adv. Rob. Syst.

[24] Arkin R.C. (1998). Behavior-based Robotics.

[25] Dissanayake G., Newman P., Clark S., Durrant-Whyte H.F., Csorba M. (2001). A solution to the simultaneous localisation and map building (SLAM) problem. IEEE T. Robotic Autom.

[26] Choset H., Lynch K., Hutchinson S., Kantor G., Burgard W., Kavraki L., Thrun S. (2005). Principles of Robot Motion: Theory, Algorithms and Implementations.

